# Rare case of Intraneural Lipoma of Digital Nerve

**DOI:** 10.1080/23320885.2021.2017779

**Published:** 2022-01-11

**Authors:** Yu-Jung Su, Laxminarayan Bhandari

**Affiliations:** Christine M Kleinert Institute of Hand and Microsurgery, Louisville, KY, USA

**Keywords:** Lipoma, digital nerve, hand

## Abstract

Lipomas, although ubiquitous, are extremely uncommon in digital nerves. We present a 68-year-old male patient with right ring finger radial digital nerve intraneural lipoma. The tumor was enucleated preserving all the nerve fascicles. We present this case to highlight the rare occurrence of lipomas within a digital nerve.

## Introduction

Schwannomas and neurofibromas constitute the commonest nerve tumors. Lipomas are rare within the substance of a nerve [1]. Among various intraneural lipomas located, Median nerve is the commonest site followed by ulnar, radial and peroneal nerves [2,3]. Intraneural lipoma of digital nerves are extremely rare with only one case reported so far. In this article, we describe a rare case of digital nerve intraneural lipoma which was successfully enucleated.

## Case

**Figure 1.**
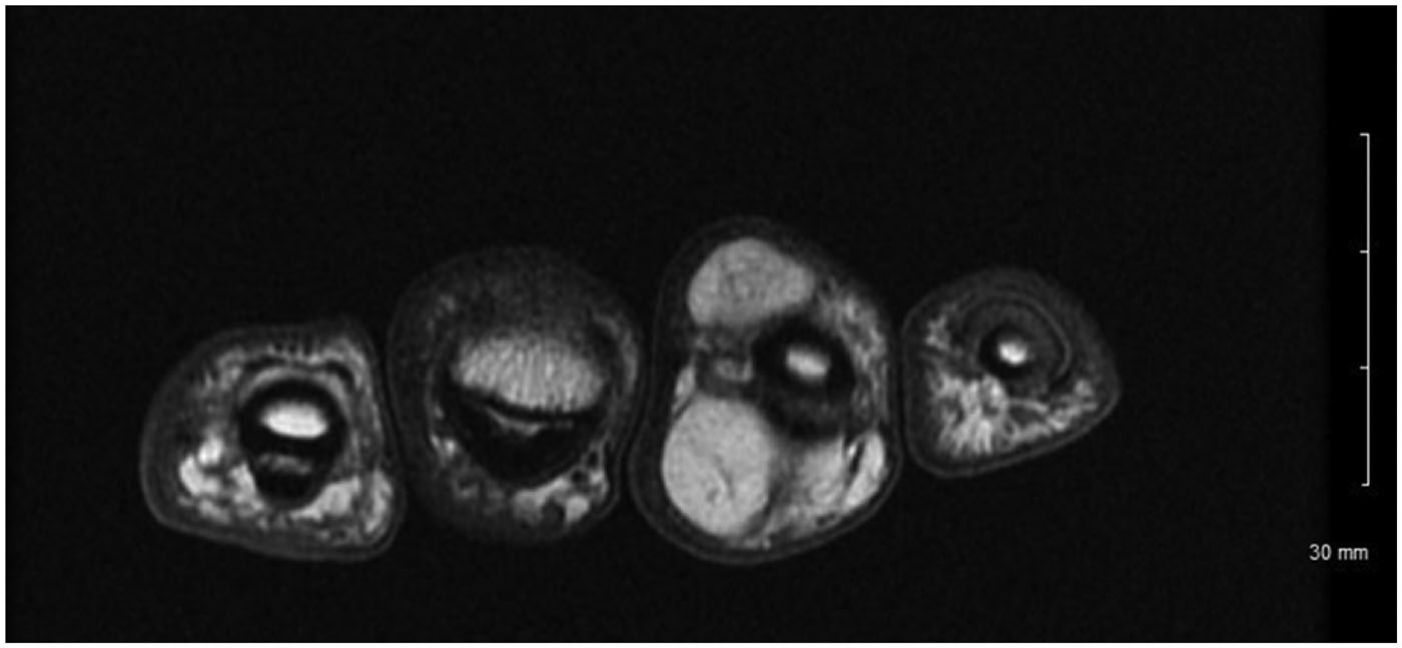
Axial MRI T1 weighted image with Fast Spin Echo (FSE) showing hyperintense lesion on both the volar and dorsal side of ring finger.

A 68-year-old man presented to our clinic with a mass on both the volar and dorsal surface of the right ring finger for 2 years. The mass was gradually enlarging. He reported no pain or numbness but had cold intolerance over the mass. Patient denied any trauma. On examination, the mass was noted to be 2 cm × 1 cm at the volar aspect of the middle phalanx. It had a dorsal extension of 1 cm × 1 cm. Right ring finger had a full range of motion. The sensation was normal with 2-point discrimination same as that of the other fingers. On palpation, the mass was firm, painless, immobile, non-translucent, non-pulsatile. There was no tinel sign. Magnetic resonance imaging demonstrated a 2.4 × 1.5 × 2.7 cm multilobulated mass with predominantly hypointense in T2-weighted images and hyperintense in T1- weighted images (Figure 1). These characteristics were suggestive of a lipoma.

**Figure 2.**
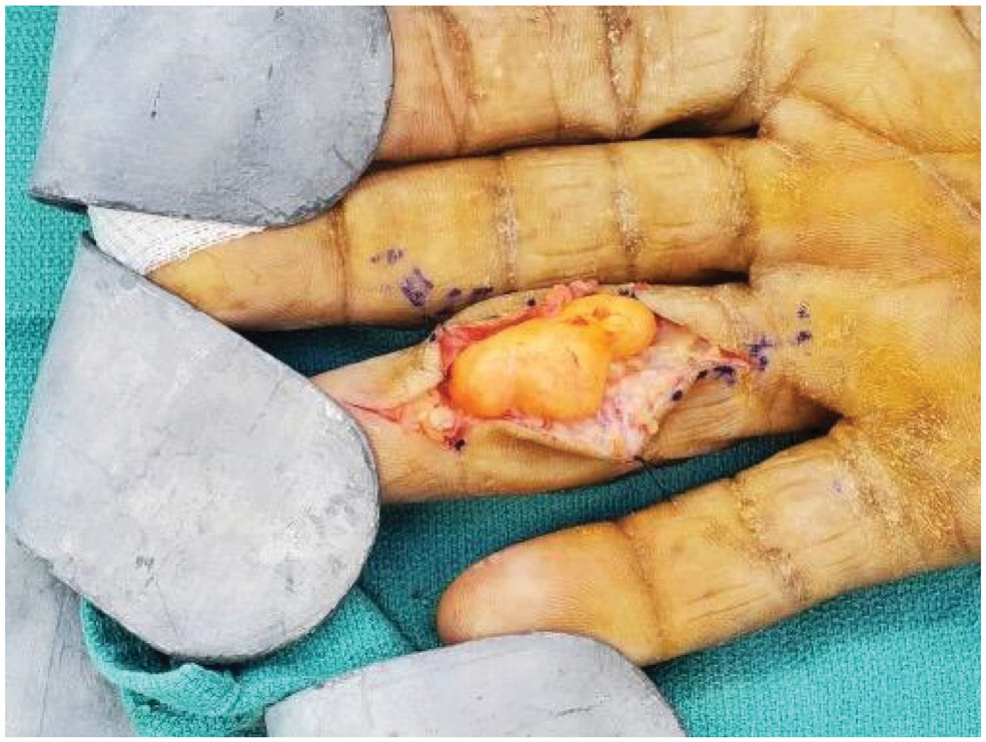
Intra operative image showing the multilobulated tumor.

**Figure 3.**
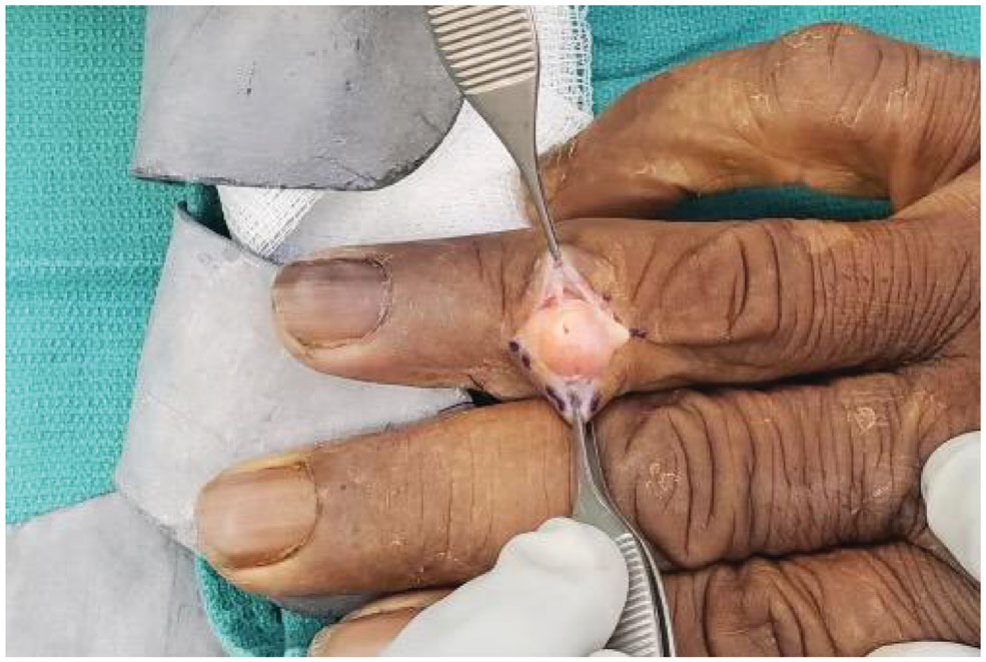
Dorsal extension of the tumor.

**Figure 4.**
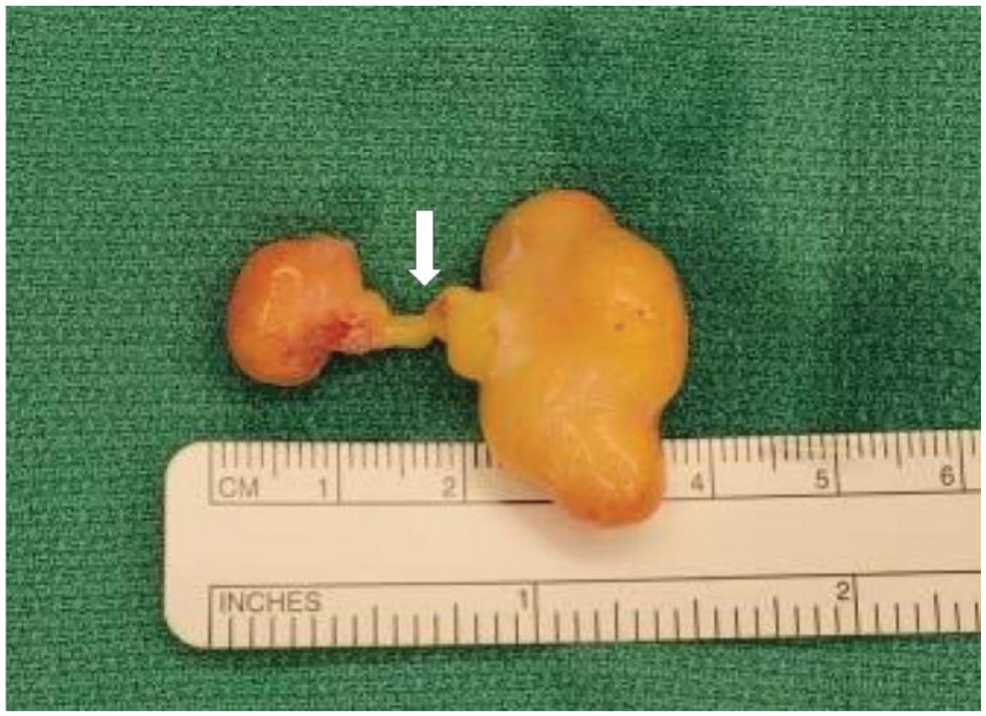
Excised specimen. The mass was divided at the middle (Arrow) .

**Figure 5.**
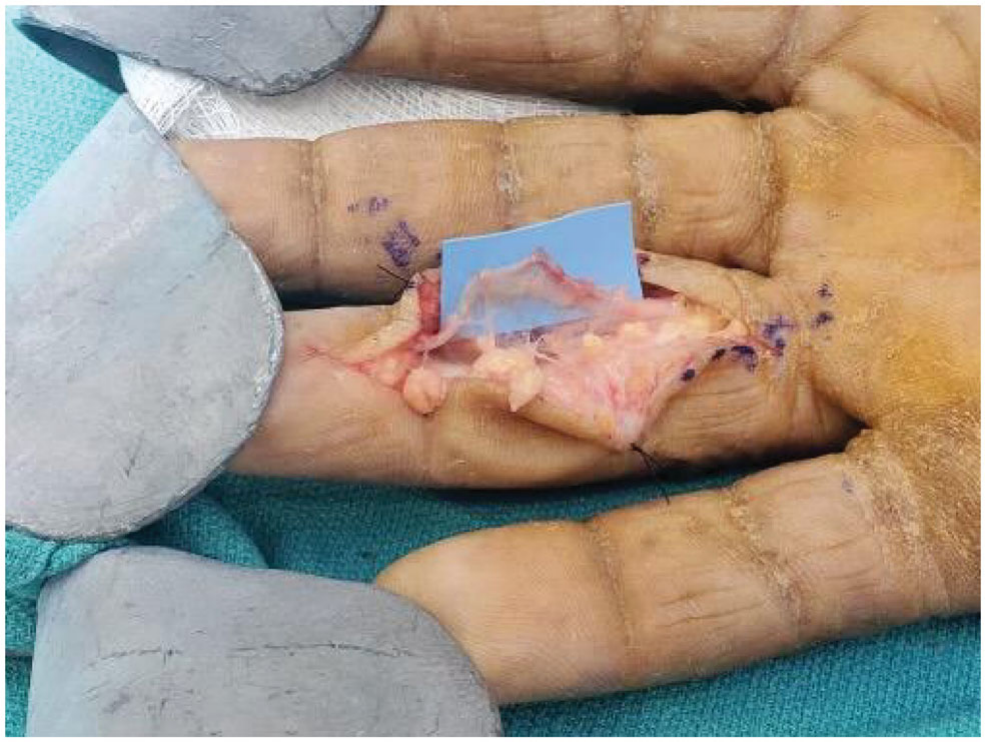
The intact nerve after excision of the tumor.

A Bruner incision was placed over the volar aspect of the right ring finger from distal interphalangeal joint to base of the finger. After lifting subcutaneous flaps, careful dissection was carried out lateral to the tendon sheath. It was noted that the mass was encapsulated with a thin flimsy sheath. The sheath was opened along the line of the neurovascular bundle (Figure 2). This flimsy sheath turned out to be the stretched out epineurium with nerve fibres. The mass was found to be communicating to its dorsal extension through an opening within the Cleland ligament . A second vertical incision was placed dorsally over the mass (Figure 3). The dorsal mass was free from surrounding tissue with no neurovascular or tendon involvement. We divided the mass midway allowing it to be removed in two pieces from volar and dorsal aspects (Figure 4). At the volar side, microdissection was performed to gently dissect fascicles away from mass, keeping the epineurium intact. The nerve was preserved (Figure 5).

Pathology revealed mature adipose tissue with focal myxoid changes suggestive lipomatosis of nerve. At 2 weeks follow up, the patient was doing well. He stated no numbness and diminishing cold intolerance. He had full range of motion and intact sensation. No recurrence was noted at 6 months follow up.

## Discussion

Although lipomas are common soft tissue tumors, fingers are not usual sites [4–6] Intraneural lipomas are extremely uncommon [4]. Intraneural lipomas arise from the growth of adipose cells within the substance of epineurium. This characteristic makes the tumor interweave with the nerve fascicles [7].

Previous reports demonstrate peripheral nerve intraneural lipomas, most commonly in the median, followed by ulnar, radial and peroneal nerves [1,8–10]. Intraneural lipomas are extremely rare in digital nerves with only one other case reported by DeSano et al. [4]. Their patient had the tumor in right ring finger, however on the ulnar side. The patient did report painand paresthesia. After surgical excision, the patient experienced some mild paresthesias along the ulnar aspect of her right ring finger, which resolved over time. The tumor was multilobulated, serpiginous 9-cm in length. In contrast, our patient, except for cold intolerance, did not have any neurological deficits. Also, in our patient the tumor was larger with extension to the dorsal aspect of the finger.

The other differential diagnoses include Schwannoma and neurofibroma. Schwannoma is the most common nerve tumor of the hand and followed by neurofibroma. Schwannomas typically present as solitary lesions and are well-encapsulated. Neurofibromas are often seen with involvement of nerve fascicles, and are difficult to dissect out of involved nerve fibres. They can be multiple as well. Clinically it is seldom possible to differentiate an intraneural lipoma from a schwannoma or neurofibroma. Imaging can aid in diagnosis. Various imaging options for digital masses include sonography, CT, MRI, bone scan etc. For soft tissue lesions MRI is preferred as it can differentiate between giant cell tumors, neurofibromas and lipomas. Additionally, MRI is superior in defining the extent of the tumor. Intra operatively, the typical appearance of lipoma becomes evident. Pathology confirms the diagnosis.

Intraneural lipomas are uncommon and extremely rare in digital nerves. Only one other case is reported in literature. Careful intraneural dissection and enucleation can preserve the nerve function. Recurrence is uncommon.

## References

[1] Marek T, Amrami KK, Mahan MA, et al. Intraneural lipomas: institutional and literature review. Acta Neurochir (Wien). 2018;160(11):2209–2218. 30242496 10.1007/s00701-018-3677-7

[2] Teles AR, Finger G, Schuster MN, et al. Peripheral nerve lipoma: case report of an intraneural lipoma of the median nerve and literature review. Asian J Neurosurg. 2016;11(4):458. 10.4103/1793-5482.181118 PMC4974996 27695575

[3] Aydin A. Carpal tunnel syndrome caused by intraneural lipoma of the median nerve and arteriovenous malformation. J Cutan Aesthet Surg. 2018;11(1):29. 29731590 10.4103/JCAS.JCAS_73_17 PMC5921447

[4] DeSano J, 2nd, Gardner P, Hart JW, et al. Intraneural lipoma of the digital nerve: a case report and literature review. Cureus. 2021;13(2):e13074. 33680616 10.7759/cureus.13074 PMC7931264

[5] Gundes H, Alici T, Sahin M. Neural fibrolipoma of the digital nerve: a case report. J Orthop Surg (Hong Kong). 2011;19(1):123. 21519094 10.1177/230949901101900129

[6] Kronberger P, Rainer C, Hittmair A, et al. Lipofibromatous hamartoma (neural fibrolipoma) of a flexor nerve of the index finger. Scand J Plast Reconstr Surg Hand Surg. 1998;32(2):237. 9646375 10.1080/02844319850158886

[7] Marek T, Spinner RJ, Syal A, et al. Strengthening the association of lipomatosis of nerve and nerve-territory overgrowth: a systematic review. J Neurosurg. 2019;132(4):1286. 30925468 10.3171/2018.12.JNS183050

[8] Balakrishnan A, Chang YJ, Elliott DA, et al. Intraneural lipoma of the ulnar nerve at the elbow: a case report and literature review. Can J Plast Surg. 2012;l20(3):e42. 23997597 PMC3433827

[9] Patel AP, Aoun SG, Al Tamimi M. Intraneural posterior interosseous nerve lipoma with complete paralysis: case report and review of the literature. Cureus. 2018;10(5):e2689. 30050744 10.7759/cureus.2689 PMC6059519

[10] Krzywosinski TB, Bingham AL, Fallat LM. Intraneural lipoma of the tibial nerve: a case report. J Foot Ankle Surg. 2017;56(1):125. 27555352 10.1053/j.jfas.2016.07.002

